# Global data on fertilizer use by crop and by country

**DOI:** 10.1038/s41597-022-01592-z

**Published:** 2022-08-17

**Authors:** Cameron I. Ludemann, Armelle Gruere, Patrick Heffer, Achim Dobermann

**Affiliations:** 1 Wageningen University & Research, Plant Production Systems, Wageningen, Netherlands; 2 International Fertilizer Association, Paris, France

**Keywords:** Plant sciences, Agriculture, Business and industry

## Abstract

Understanding how much inorganic fertilizer (referred to as fertilizer) is applied to different crops at national, regional and global levels is an essential component of fertilizer consumption analysis and demand projection. Good information on fertilizer use by crop (FUBC) is rarely available because it is difficult to collect and time-consuming to process and validate. To fill this gap, a first global FUBC report was published in 1992 for the 1990/1991 period, based on an expert survey conducted jointly by the Food and Agriculture Organization (FAO) of the UN, the International Fertilizer Development Center (IFDC) and the International Fertilizer Association (IFA). Since then, similar expert surveys have been carried out and published every two to four years in the main fertilizer-consuming countries. Since 2008 IFA has led these efforts and, to our knowledge, remains the only globally available data set on FUBC. This dataset includes data (in CSV format) from a survey carried out by IFA to represent the 2017–18 period as well as a collation of all historic FUBC data.

## Abstract (summary)

**Table:** 

Measurement(s)	Fertilizer use by crop and country for nitrogen, phosphorus and potassium.
Technology Type(s)	Survey
Factor Type(s)	Crop and country
Sample Characteristic - Organism	Plantae
Sample Characteristic - Environment	World
Sample Characteristic - Location	World

## Background & Summary

Fertilizer often constitutes the major source of nutrients in a crop system^1,2^. Therefore the input of nutrients in the form of fertilizer is often an important component of crop nutrient balances and assessments or monitoring of nutrient use efficiency at different scales^2^. Crop nutrient balances highlight regions of the world where crop production could be limited by nutrients and/or where there are an excessive quantity being applied. The former situation may result in poor crop or livestock production with detrimental effects on food security, while the latter situation may lead to a loss of nutrients with potentially detrimental effects on the environment.

Collection of FUBC data is difficult and time-consuming at a global scale. While statistics for grain production in a country can be relatively easily estimated given you can attribute the quantity of grain back to a certain crop, this is not the case for FUBC data. Firstly, good information on country level FUBC is rarely available or monitored by statistical bureaus. Secondly, attributing fertilizer back to one certain crop can be made more difficult in countries where there is integration of grassland with crop production. In these cases fertilization of arable land can be part of a crop rotation that aids productivity of the subsequent grassland crops, or vice versa. Thirdly, multiple crops per year, or multiple crops in the same area of land can make a binary distribution of FUBC back to a single crop more difficult.

The first country level FUBC data were published in 1992 to fill the gap in data availability^3^ with (up until the present study) the latest results published in 2017^4^. The first data were based on a survey of experts conducted by FAO, IFDC and IFA. Similar surveys were undertaken and published every two to four years for the main fertilizer-consuming countries (Table 1). IFA has led these survey efforts since 2008. To our knowledge these are the only globally available dataset for FUBC.

**Table 1:** Years represented in each fertilizer use by crop (FUBC) report used in the combined dataset.

FUBC report number	Reference	Year(s) represented in each report*
1	FAO, *et al*.^3^	1984, 1986,1987,1988,1989,1990,1991
2	FAO, *et al*.^9^	1978,1989/90,1990,1990/91,1991,1991/92,1992,1992/93,1993
3	FAO, *et al*.^10^	1986,1987,1989,1990,1991,1992,1993,1994,1995
4	FAO, *et al*.^11^	1986,1987,1989,1990,1991,1992,1993,1994,1995,1996,1997
5	FAO, *et al*.^12^	1994,1995,1996,1997,1997/98,1998,1998/99,1999,1999/2000,2000,2001
6	Heffer^13^	2006, 2006/07, 2007 2007/08
7	Heffer^14^	2010, 2010/11
8	Heffer, *et al*.^4^	2014, 2014/15

* In some cases there were differences in the categorization of periods used, with some representing calendar years, and others representing fertilizer years (e.g. 1989/90).

The current dataset includes data from the latest FUBC survey conducted by IFA as well as a collation of all available FUBC data shown in Table 1. The current survey benefited from the agronomic expertise of numerous experts to provide and validate estimates. This is the best effort that IFA can achieve with its current resources. It provides a general overview of how fertilizers are being used worldwide for the three main nutrients: nitrogen (N), phosphorus (P, reported here as P_2_O_5_) and potassium (K, reported here as K_2_O). We caution, however, that for many countries the estimates provided here are associated with substantial uncertainties. Likewise, comparisons with previous reports must be made with caution because methodologies and sources of information have changed over time. Nevertheless, this dataset constitutes the best estimates of FUBC at a country level with a global coverage to date.

## Methods

### Data acquisition

Data were collated from previously published FUBC surveys (referred herein, as historic FUBC data), as well as data from the latest IFA FUBC survey (referred herein, as the latest FUBC data).

### Data acquisition from historic (published) data

The historic FUBC data were converted into comma-separated values (CSV) file format by first converting the pdf reports into Microsoft Excel (version 2102, Microsoft Corporation, Redmond Washington, USA) format using online software from www.adobe.com. The excel document was visually checked for errors and corrected where necessary before it was saved as a CSV file. The original country names from the reports were kept in the final datafile as the “Original_country_name_in_FUBC_report” parameter (Tables 2, 3). The “Country” and “ISO3_code” parameter information included the names which had been converted into the standardised full country names and alpha-3 code formats respectively following the ISO 3166 international standards (https://www.iso.org/publication/PUB500001.html). The “Region_IFA” parameter information was based on the country categorisation listed in Supplementary Table 1. Crop names were kept the same as those reported in the original publication, and any missing values were assigned NA.

**Table 2:** List of parameters, formats and units for the fertilizer use by crop (FUBC) data in the combined dataset (which included all available FUBC surveys, and had the latest survey results where values had been aggregated to crop categories that aligned to the last published FUBC report^4^).

Parameter	Description	Format	Units
Original_country_name_in_FUBC_report	Original name of country used in FUBC report	Character	Character
Country	Country name based on official United Nations English name, with the exception that references to Belgium-Luxembourg were converted to ‘Belgium’, and referrals to Taiwan were converted to China, Taiwan	Character	Character
ISO3_code	The 3-letter ISO3 United Nations code to signify country or region. Note that China, Taiwan was given the TWN 3-letter code	Character	Character
Region_IFA	Region, based on the International Fertilizer Association (IFA) list of aggregate countries and regions	Character	Character
Year	Year in which the data relates to. Year is in character format because in some reports the data relate to non-calendar years e.g. 1991/92, 1997–98. These therefore include a mixture of calendar and ‘fertilizer’ years	Character	Character
FUBC_report_number	The fertilizer use by crop (FUBC) report number. This is a sequential number assigned to each report since they were first published	Numeric	Integer
Year_FUBC_publication	The fertilizer use by crop (FUBC) report, year of publication	Numeric	Integer
Crop	Crop type, based on those originally reported in the fertilizer use by crop (FUBC) reports	Character	Character
Crop_area_k_ha	The total crop area which may include planted or harvested areas depending on what data the survey respondents had available	Numeric	Kilo hectares (1000* ha/year)
N_k_t	Total nitrogen applied to total crop area	Numeric	Kilo (*1000) metric tonnes of elemental nitrogen/year
P2O5_k_t	Total P_2_O_5_ applied to total crop area	Numeric	Kilo (*1000) metric tonnes of P_2_O_5_/year
K2O_k_t	Total K_2_O applied to total crop area	Numeric	Kilo (*1000) metric tonnes of K_2_O/year

**Table 3:** List of parameters, formats and units for the fertilizer use by crop (FUBC) data in the combined dataset (which included all available FUBC surveys, and had the latest survey results where values had been aggregated to crop categories that aligned to the last published FUBC report^4^) (continued).

Parameter	Description	Format	Units
N_P2O5_K2O_k_t	Total nitrogen + P_2_O_5_ + K_2_O applied to total crop area	Numeric	Kilo (*1000) metric tonnes of N + P_2_O_5_ + K_2_O/year
N_rate_kg_ha	Mean application rate of nitrogen to area of crop that actually received fertilizer (see N_pc_fert for percentage of crop area where N was applied)	Numeric	Kilograms of nitrogen per hectare (kg N/ha/year)
P2O5_rate_kg_ha	Mean application rate of P_2_O_5_ to area of crop that actually received fertilizer (see P_2_O_5__pc_fert for percentage of crop area where P_2_O_5_ was applied)	Numeric	Kilograms of P_2_O_5_ per hectare (kg P_2_O_5_/ha/year)
K2O_rate_kg_ha	Mean application rate of K_2_O to area of crop that actually received fertilizer (see K_2_O_pc_fert for percentage of crop area where K_2_O was applied)	Numeric	Kilograms of K_2_O per hectare (kg K_2_O /ha/year)
N_pc_fert	Percentage of total crop area that received any nitrogen fertilizer	Numeric	Percent (%)
P2O5_pc_fert	Percentage of total crop area that received any P_2_O_5_ fertilizer	Numeric	Percent (%)
K2O_pc_fert	Percentage of total crop area that received any K_2_O fertilizer	Numeric	Percent (%)
Aver_N_rate_kg_ha	Mean application rate of nitrogen across total crop area	Numeric	Kilograms of nitrogen per hectare (kg N/ha/year)
Aver_P2O5_rate_kg_ha	Mean application rate of P_2_O_5_ across total crop area	Numeric	Kilograms of P_2_O_5_ per hectare (kg P_2_O_5_/ha/year)
Aver_K2O_rate_kg_ha	Mean application rate of K_2_O across total crop area.	Numeric	Kilograms of K_2_O per hectare (kg K_2_O /ha/year)
Aver_N_P2O5_K2O_rate_kg_ha	Mean application rate of nitrogen + P_2_O_5_ + K_2_O across total crop area	Numeric	Kilograms of N + P_2_O_5_ + K_2_O per hectare (kg N + P_2_O_5_ + K_2_O /ha/year)

### Data acquisition from latest survey

The latest FUBC data were collated from a survey of IFA’s country correspondents, similar to the methodology employed in the previous IFA FUBC assessment^4^. Countries were selected for inclusion in this survey based on relative contribution to global fertilizer use with countries included in the survey representing over 90% of global fertilizer use. The survey was carried out between 2020 and 2022. The questionnaire or specific questions were sent to 88 persons, groups of persons or organizations (Supplementary Table 2), covering 76 countries and multi-country estimates for a few specific crops. Data and/or information were received from 32 persons (or groups of persons or organizations) for 63 countries and used for 53 countries. In most cases one to two respondents provided information for each country at the national administrative level (see Acknowledgements section for further details). However, the exception to this was for European Union (EU) countries where only five respondents provided estimates for all the EU countries. In cases where there were conflicts in estimates between correspondents, the correspondents were contacted to understand where the differences came from. The estimates were compared with estimates of total fertilizer use by country received from a separate IFA survey (https://www.ifastat.org/databases/plant-nutrition, referred herein as IFASTAT) to ensure there was general agreement in values. Where the estimates of total fertilizer use by country from these two different surveys did not align, efforts were made to understand why there were these differences. In particular, the areas of crop suggested by the survey respondents were compared with FAOSTAT^5^ values and in some cases (particularly China as is described in the Technical validation section) non-FAOSTAT areas for the crop were used to get an estimate of total fertilizer use in that country that seemed justifiable based on expert opinion from the authors of this dataset.

The questionnaire itself was composed of an Excel (version 2102, Microsoft Corporation, Redmond Washington, USA) spreadsheet (Supplementary Table 3). Rows were included in the spreadsheet for the correspondent to add estimates of the area of crop, percentage of total crop area that had been fertilized, and total application of N, P_2_O_5_ and K_2_O for each crop for their country of expertise. The exact methodology used by respondents and statistical agencies to develop estimates of fertilizer use by crop is not known to the authors. Methodologies therefore may differ across countries and may range from including actual farm survey data to simply estimating representative application rates based on their knowledge of the country. Survey participants were asked to report planted area for each crop, however in some cases harvested area was provided. If these data were not available, the FAOSTAT^5^ harvested areas for each crop were used. Planted areas were used for 51 countries, harvested areas were used for 11 countries and ‘fertilized area’ was used for one country (Ukraine).

### Processing of latest survey data

In contrast to the historic data included in the combined dataset, (where we kept the same crop names as were used in the published reports), data from the latest survey were aggregated, where possible, into a limited set of aggregate crop names (see Table 4). This was performed to align to the crop categories reported by Heffer, *et al*.^4,6^. However, this resulted in some of the information from the original survey being lost. Therefore, in addition to the combined dataset, we also include a separate raw datafile of the latest survey results that include information for the original crop names. The R code required to convert the raw data file for the latest survey results and merge it into the combined csv file is available at: https://github.com/ludemannc/FUBC_1_to_9_2022.git.

**Table 4:** Categorisation of crop names for the latest* fertilizer use by crop survey data.

Final crop name in datafile	Variations of crop names (from survey respondents) included in final crop name category.
Wheat	Wheat
Rice	Rice
Maize (grain)	Crop names that either stated maize only, or mentioned maize and grain
Maize green	Crop names that mentioned maize and the fact it was not used for grain (e.g. Maize green, maize silage and maize bioenergy)
Other cereals	Cereal, irrigated field crop, other cereals, grain mixed sheep & beef
Soybeans	Soybeans
Pulses	Pulses, beans, peas, black-eyed peas (niébé)
Oil palm	Oil palm, oil palm fruit
Rapeseed	Canola, rapeseed, oilseed rape
Other oil crops	Coconut, sunflower, other oil crops, other oil seeds, linseed, sesame, groundnut, peanut, olive
Cottonseed	Cotton
Sugar beet	Sugar beet
Sugar cane	Sugar cane
Tea	Tea
Coffee green	Coffee, green
Roots/tubers	Roots and tubers, potato, taro, cocoyam, cassava, yam
Fruits and treenuts	Fruits, melon, citrus, treenuts
Vegetables	Vegetables, tomato, onion, sweet corn, garlic
Grassland	Grass, pasture, hay, rangeland, lucerne, perennial crops, fodder, forages
Residual (Other crops)	Forest, rubber, flowers, cocoa, fresh herbs, orchards, amenity horticulture, ginger, horticulture, tobacco, other industrial crops, other crops, short rotation coppice, perennial herbaceous crops (Miscanthus, etc.), residual

*Crop information for the historic fertilizer use by crop data were not changed from that reported in the original source.

In relation to the latest survey data that had been aggregated by crop names (in the combined data file), in some cases it was not possible to categorize the crop names received from the survey respondents into the categories listed in Table 4. For instance, for most countries in the European Union, the data followed Fertilizers Europe’s own method of categorisation whereby rice was grouped with rye, triticale and oats, and soybeans were grouped with sunflower and linseed. For countries in the European Union, rice therefore had to be categorized as an ‘Other cereal’, while soybeans had to be categorized as an ‘Other oil crop’. Respondents from New Zealand grouped wheat, oats and barley into an aggregated cereal grains category. In this case the cereal grains category data had to be included in the ‘Other cereal’ crop category. The total nutrients applied to each (aggregate) crop category in each country were divided by the total area of each (aggregate) crop category in each country to estimate the mean application rates for each nutrient per hectare.

Estimates for the most recent survey could not be obtained for ten important fertilizer consuming countries: Australia, Bangladesh, Egypt, Indonesia, Iran, Malaysia, Mexico, Morocco, Russia and Vietnam. For these countries, the previous average application rates (calculated based on nutrient quantities and FAO 2014 crop area) were multiplied by 2018 FAO (harvested) crop area. The resulting nutrient quantities were adjusted so that total fertilizer use in the country matched the consumption of nutrients estimated by country from IFASTAT. For example, if the 2014 application rates were multiplied by the 2018 FAO crop areas and this resulted in a total nutrient consumption for a certain country that needed 10% more to be equivalent to the value from IFASTAT then the 2014 application rates were multiplied by 1.1 to get alignment in total nutrient consumption between the two surveys.

## Data Records

### Combined dataset (FUBC_1_to_9.csv)

This dataset can be downloaded from DRYAD^7^ (10.5061/dryad.2rbnzs7qh) and is available in CSV file format. Tables 2, 3 provide an overview of the data records in the combined data file.

*Original_country_name_in_FUBC_report*, *Country*, *ISO3_code*, and *Region_IFA* have been well described in the Methods section and are included as names in text format. *Year* data are not represented as integers because in some cases non-integer calendar years were used e.g. 1991/92 and 1997/98. *FUBC_report_number* indicates the integer assigned to each FUBC report in temporal order of publication. *Year_FUBC_publication* indicates the year (as an integer) in which the FUBC report was published. In the historic component of the FUBC data, the *Crop* information is the same as that published in the original FUBC report. In the latest FUBC survey data the original crop names used by respondents of the survey were categorized following the method described in the *Methods* section. *Crop_area_k_ha* was the total crop area in thousands of hectares (either as planted or harvested area) as a numeric value depending on what the survey participant had available. Unfortunately, it was not possible to differentiate whether each crop area estimate referred to planted or harvested area.

*N_k_t, P2O5_K_t* and *K2O_k_t* reflected the total quantities of nitrogen, P_2_O_5_ and K_2_O respectively in thousands of metric tonnes per year. In some cases the total quantities of nutrients do not equal the total crop areas multiplied by the percentage of total crop area that received fertilizer and the kilograms of nutrient rate per hectare. This is because, firstly the latest FUBC data did not include percentages of total crop area that had been fertilized. The values for nutrient application rates were means for across total crop area in the latest FUBC data. Secondly, the references for the historic FUBC data (see Table 1) warned that in some cases values did not add up due to rounding errors. Numeric values from the historic FUBC data were kept the same as those reported in the original publications. *N_ P2O5_K2O_k_t* is the sum of the *N_k_t, P2O5_K_t* and *K2O_k_t* numeric values.

*N_rate_kg_ha, P2O5_rate_kg_ha* and *K2O_rate_kg_ha* are the mean application rates of nitrogen, P_2_O_5_ and K_2_O respectively in kilograms of nutrient per hectare per year. These are the mean application rates of each respective nutrient to the areas of crop that actually received any fertilizer. *N_pc_fert, P2O5_pc_fert, K2O_pc_fert* denote in numeric terms the percentage of total crop area that has had any nitrogen, P_2_O_5_ or K_2_O fertilizer applied to it respectively.

*Aver_N_rate_kg_ha, Aver_P2O5_rate_kg_ha, Aver_K2O_rate_kg_ha*, and *Aver_N_P2O5_K2O_rate_kg_ha*, are the average application rates of nitrogen, P_2_O_5_, K_2_O and N + P_2_O_5_ + K_2_O respectively (in kg per hectare per year of the respective nutrients) across the total crop areas. They are a function of the total nutrients applied divided by the total crop area. Note that in the original sources of historic FUBC data there was variation between countries, crops and years as to whether the average application rates across total crop area, and/or average application rates across fertilized areas of crops were used. These numeric data were included in the dataset as they were reported in the original publication, and no attempt was made to interpolate data to fill in missing values.

A meta data file accompanies the combined data file and is named Meta_data_FUBC_1_to_9.csv.

### Latest FUBC survey raw data file (FUBC_9_raw_data.csv)

This dataset can be downloaded from DRYAD^7^ (10.5061/dryad.2rbnzs7qh) and is available in CSV file format. Details of each parameter in this file are listed in Table 5.

**Table 5:** List of parameters, formats and units for the latest fertilizer use by crop raw data file (FUBC_9_raw_data.csv) where original crop information was retained.

Parameter	Description	Format	Units
Original_country_name_in_FUBC_report	Original name of country used in FUBC report	Character	Character
Original_crop_name_in_FUBC_report	Crop type, based on those originally reported in the fertilizer use by crop (FUBC) reports	Character	Character
Year	Year the fertilizer application information relates to (this is in character format as it includes some crops that grow across multiple years, such as 2017/18 which denotes a fertilizer year starting in mid 2017 and ending in mid 2018)	Character	Character
Year_for_FAO_area	Food and Agriculture (FAO) Year that is most applicable to the year in which the fertilizer application information estimation was made for	Numeric	Integer to denote Gregorian calendar years
FAO_area_ha	Food and Agriculture (FAO) area for each crop	Numeric	Hectares per year
IFA_area_ha	International Fertilizer Association (IFA) area for each crop, based on Fertilizer Use By Crop (FUBC) survey information	Numeric	Hectares per year
FAO_area_used_Yes_No	Provides an indication of whether it was decided the FAO or the IFA estimate of crop area is to be used, whereby Yes = FAO crop are used, and No = IFA crop area is used	Character	Character
IFA_N_t	Total nitrogen applied to total crop area per year	Numeric	Metric tonnes
IFA_P2O5_t	Total P_2_O_5_ applied to total crop area per year	Numeric	Metric tonnes
IFA_K2O_t	Total K_2_O applied to total crop area per year	Numeric	Metric tonnes
Comment	Comments made about each data point	Character	Character
Survey_respondent	Information on whose information (from the survey) was used in the estimate	Character	Character

*Original_country_name_in_FUBC_report* and *Original_crop_name_in_FUBC_report* provide information on the original country and crop name from the survey respectively as names in text format. *Year* data are not represented as integers, but are instead represented as text to align to the format of *Year* in the combined (FUBC_1_to_9.csv) dataset. *Year_for_FAO_area* indicates the most appropriate FAO ‘year’ for aligning FUBC survey data with FAO crop areas and are represented as integer values. The *FAO_area_ha* and *IFA_area_ha* represent the numeric areas of crop per year based on the FAO data^5^ and the latest FUBC survey respectively.

FAO_area_used_Yes_No indicates whether the FAO or latest FUBC survey estimates of crop are were used in character format as either a ‘Yes’ or ‘No’. The *IFA_N_t, IFA_P2O5_t*, and *IFA_K2O_t* indicate in numeric terms the total quantity (in metric tonnes) of nutrient applied to the total crop area for a country in a year.

*Comment* and *Survey_respondent* provides miscellaneous information (in character format) related to data that came from the survey as well as information on whose information was used in the estimate, respectively.

A meta data file accompanies the latest FUBC survey dataset raw data file and is named Meta_data_ FUBC_9_raw_data.csv.

### IFA regions data file (IFA_Regions.csv)

This dataset can be downloaded from DRYAD^7^ (10.5061/dryad.2rbnzs7qh) and is available in CSV file format. This file indicates how countries were assigned to ‘IFA Regions’. It includes columns of information (all in character format) for *Country*, ISO3_Code, and *Region_IFA*. *Country* indicates the country name based on the official United Nations English name, *ISO3_Code* indicates the 3-letter ISO3 United Nations code to signify country or region, and *Region_IFA* indicates categories of regions based on the International Fertilizer Association (IFA) list of aggregate countries and regions.

A meta data file accompanies the IFA_Regions data file and is named Meta_data_ IFA_Regions.csv.

### Country tables data file (Country_tables.xlsx)

This dataset can be downloaded from DRYAD^7^ (10.5061/dryad.2rbnzs7qh) and is available in xlsx (Excel) file format. Country_tables.xlsx contains the planted areas (in hectares), total nutrients applied, nutrients applied per hectare and the percentage share of nutrient use for nitrogen, P_2_O_5_, and K_2_O for the different categories of crops, for all countries where data were available. The categories of crops in this file are the same as those reported by respondents in the survey.

## Technical Validation

This is the only known world-wide survey of fertilizer use by crop at a country level, which makes validation of the nutrient application rate data within this dataset difficult. As mentioned in the Method section, respondents of the survey used different methodologies for making their estimates and the crop categories also varied somewhat by country. Where possible, estimates from the respondents were compared with official statistics from the respective country and/or from FAOSTAT^5^. In some cases where there were major discrepancies, respondents from the survey were contacted to clarify their estimates. The main forms of clarification included correcting obvious mistakes in either quantities of fertilizer or areas of crop, clarifying the types of crops or grassland considered, clarification of production practices that helped explain higher than expected fertilizer application rates (for example, application rates of irrigated area are greater than those on rainfed areas). If the respondent could not provide an explanation, estimates from other sources were used. If better estimates were not available the following three options were utilised: (1) if the crop area was small relative to the country’s total crop area, the crop was attributed to the residual category, (2) if the crop area accounted for a significant share of the country’s total, and the country was a small fertilizer consumer, we decided not to publish the country data, and (3) if the crop area accounted for a significant share of the country’s total, and the country was a significant consumer of fertilizers, then an estimate was made based on an extrapolation of the data from the previous survey as previously described in the ‘Processing of latest survey data’ section.

Furthermore, total nutrient use for each country was estimated through multiplication of the application rate per hectare by the area of each crop. This allowed estimates of total nutrient use for each country to be compared with estimates of total nutrient consumption by country from IFASTAT. As shown in Supplementary Table 4, there was general agreement in the total quantities of nitrogen, P_2_O_5_ and K_2_O (applied as fertilizer) for each country depending on whether it was based on the IFASTAT data or data from the latest FUBC survey. This is indicated by the relatively low percentage differences in total values. There were some countries where there was substantial variation between the two estimates of total fertilizer application per country. The countries with the greatest variation included Senegal (72% difference), followed by Paraguay (63% difference) and Cyprus (46% difference). To put the contribution of these countries’ fertilizer consumption into perspective, out of the 3 countries listed, Paraguay has the greatest total contribution to world consumption of the three main nutrients. Total annual consumption of nitrogen, P_2_O_5_ and K_2_O as inorganic fertilizer for Paraguay made up only 0.3% of total world consumption of those nutrients. Therefore the countries with most variation in estimates will have an insignificant effect on estimates of nutrient consumption at the global aggregate level. Nevertheless, these sources of variation represent considerable uncertainty for the estimates of those countries. Given that we did not receive information on how each survey respondent made their estimates, we were not able to interrogate any further why this variation may have occurred and should be an area of focus in any subsequent surveys. We can only speculate that errors in the methods by which the estimates were made for the IFASTAT and/or latest survey results (for example, if some crops are missing) contributed to this variation. The R code used for the aforementioned comparison in estimates using results from IFASTAT and the latest survey results is available at: https://github.com/ludemannc/FUBC_1_to_9_2022.

Estimates for some countries are therefore associated with substantial uncertainties. Therefore analysis of these data across years and between countries should be made with caution. Likewise, comparisons with previous reports must be made with caution because methodologies, crop categories and sources of information have changed over time. This is especially the case for China. China did not have the greatest difference in final estimates of fertilizer consumption between IFASTAT and the latest FUBC survey compared with other countries (it had 35% variation). However, China is a major consumer of fertilizer, with 32% of the world’s use of fertilizer (as nitrogen + P_2_O_5_ + K_2_O) coming from this country. Any variation in consumption by China will have a significant effect on estimates of total world consumption of fertilizer. Unfortunately, the data for China are difficult to reconcile with the FAOSTAT^5^ published crop harvest area statistics and IFASTAT consumption estimates. Average 2018 fertilizer application rates were collected for the major crops of China based on county-level statistics published by the National Development and Reform Commission of the People’s Republic of China (NDRC), but also farm survey data collected by research groups at China Agricultural University (CAU) and the Chinese Academy of Agricultural Sciences (CAAS). However, when multiplied with 2018 FAO crop area estimates, the resulting 2018 fertilizer consumption for the country exceeded the IFASTAT total national consumption estimate by 42%, and that of the National Bureau of Statistics by 15%. The gap would be even larger if average fertilizer application rates could also be obtained for minor crops. The IFASTAT estimate of total fertilizer use in China is obtained by adding apparent consumption across all fertilizer products (apparent consumption is the sum of production, imports and beginning stocks, minus exports and ending stocks). It is possible that the IFASTAT estimate of total consumption in China is lower than actual fertilizer consumption but it is not expected that the actual fertilizer consumption would be 42% greater than the IFASTAT estimates.

The 42% difference could be explained by a combination of reasons: overestimated crop area, overestimated average fertilizer application rates (if less than 100% of crop area is fertilized), and/or underestimated apparent consumption. At this stage, we are not able to resolve these differences. However, considering the huge importance of the major cereal crops and the generally known uncertainties about crop harvested area statistics in China, we replaced the 2018 FAO crop area estimates for rice, wheat and maize with more accurate estimates based on integrating multi-data sources, including remote sensing from Luo *et al*.^8^. This adjustment reduced the consumption estimate difference to about 35% compared to IFASTAT (see Supplementary Table 4).

## Usage Notes

The following files are available at the DRYAD repository^7^ (10.5061/dryad.2rbnzs7qh):

- README_FUBC_DATA_2022.txt
  - Provides a summary of all the datafiles in the DRYAD repository.
- FUBC_1_to_9.csv
  - Includes fertilizer use by crop data from the 8 previously published reports (FUBC 1 to 8), as well as the fertilizer use data from the latest (9^th^) survey (FUBC 9) carried out by the International Fertilizer Association. It is important to note that the FUBC 9 data have been aggregated by crop categories that align to those used in the previous survey (FUBC 8).
  - In total the combined dataset included 516 unique crop names, many of which have only slights variations in spelling based on how they were written in the previously published FUBC reports.
- Meta_data_FUBC_1_to_9.csv
  - Includes meta-data associated with the FUBC_1_to_9.csv file.
- FUBC_9_raw_data.csv
  - Includes fertilizer use by crop data from the latest fertilizer use by crop survey.
  - This file includes data where the crop names remained the same as those originally received by survey respondents. This file can be used to access the original crop information (for FUBC 9) that is otherwise lost from the FUBC_1_to_9.csv file due to crop aggregation.
  - In total this file contains 159 unique crop names.
- Meta_data_FUBC_9_raw_data.csv
  - Includes meta-data associated with the FUBC_9_raw_data.csv file.
- IFA_Regions.csv
  - Includes information shown in Supplementary Table 1 as a csv file for programmatic ease of use.
- Meta_data_IFA_Regions.csv
  - Includes meta-data associated with the IFA_Regions.csv file.
- Country_tables.xlsx
  - Includes original fertilizer use by crop data from the latest survey with separate excel worksheets for each country. This file includes more information (as ‘Notes’) on how estimates were made for each crop and country.

## Supplementary information

Supplementary Information

## Data Availability

The R project associated with aggregating the raw datafile (FUBC_9_raw_data.csv) into crop categories for inclusion in the combined data file (FUBC_1_to_9.csv), and creation of results in Supplementary Table 4 are available at https://github.com/ludemannc/FUBC_1_to_9_2022.
