# Supplementary material for: Global data on fertilizer use by crop and by country
Source: Sci Data. 2022 Aug 17;9:501. doi: 10.1038/s41597-022-01592-z (PMC9385665; doi:10.1038/s41597-022-01592-z)
Supplement: Supplementary file 1 — Supplementary Information [file 41597_2022_1592_MOESM1_ESM.docx]

### Supplementary Table 1: IFA regions for each Country and Alpha-3 code (ISO3 code)

| **Country** | **ISO3 code** | **Region IFA** |
| --- | --- | --- |
| Afghanistan | AFG | West Asia |
| Albania | ALB | Western and Central Europe |
| Algeria | DZA | Africa |
| Andorra | AND | Western and Central Europe |
| Angola | AGO | Africa |
| Antigua and Barbuda | ATG | Latin America |
| Argentina | ARG | Latin America |
| Armenia | ARM | Eastern Europe and Central Asia |
| Australia | AUS | Oceania |
| Austria | AUT | Western and Central Europe |
| Azerbaijan | AZE | Eastern Europe and Central Asia |
| Bahamas | BHS | Latin America |
| Bahrain | BHR | West Asia |
| Bangladesh | BGD | South Asia |
| Barbados | BRB | Latin America |
| Belarus | BLR | Eastern Europe and Central Asia |
| Belgium | BEL | Western and Central Europe |
| Belize | BLZ | Latin America |
| Benin | BEN | Africa |
| Bhutan | BTN | East Asia |
| Bolivia (Plurinational State of) | BOL | Latin America |
| Bosnia and Herzegovina | BIH | Western and Central Europe |
| Botswana | BWA | Africa |
| Brazil | BRA | Latin America |
| Brunei Darussalam | BRN | East Asia |
| Bulgaria | BGR | Western and Central Europe |
| Burkina Faso | BFA | Africa |
| Burundi | BDI | Africa |
| Cabo Verde | CPV | Africa |
| Cambodia | KHM | East Asia |
| Cameroon | CMR | Africa |
| Canada | CAN | North America |
| Central African Republic | CAF | Africa |
| Chad | TCD | Africa |
| Chile | CHL | Latin America |
| China | CHN | East Asia |
| China, Taiwan | TWN | East Asia |
| Colombia | COL | Latin America |
| Comoros | COM | Africa |
| Congo | COG | Africa |
| Costa Rica | CRI | Latin America |
| Côte D'Ivoire | CIV | Africa |
| Croatia | HRV | Western and Central Europe |
| Cuba | CUB | Latin America |
| Cyprus | CYP | West Asia |
| Czech Republic | CZE | Western and Central Europe |
| Democratic People's Republic of Korea | PRK | East Asia |
| Democratic Republic of the Congo | COD | Africa |
| Denmark | DNK | Western and Central Europe |
| Djibouti | DJI | Africa |
| Dominica | DMA | Latin America |
| Dominican Republic | DOM | Latin America |
| Ecuador | ECU | Latin America |
| Egypt | EGY | Africa |
| El Salvador | SLV | Latin America |
| Equatorial Guinea | GNQ | Africa |
| Eritrea | ERI | Africa |
| Estonia | EST | Eastern Europe and Central Asia |
| Eswatini | SWZ | Africa |
| Ethiopia | ETH | Africa |
| Fiji | FJI | Oceania |
| Finland | FIN | Western and Central Europe |
| France | FRA | Western and Central Europe |
| Gabon | GAB | Africa |
| Gambia (Republic of The) | GMB | Africa |
| Georgia | GEO | Eastern Europe and Central Asia |
| Germany | DEU | Western and Central Europe |
| Ghana | GHA | Africa |
| Greece | GRC | Western and Central Europe |
| Grenada | GRD | Latin America |
| Guatemala | GTM | Latin America |
| Guinea | GIN | Africa |
| Guinea Bissau | GNB | Africa |
| Guyana | GUY | Latin America |
| Haiti | HTI | Latin America |
| Honduras | HND | Latin America |
| Hungary | HUN | Western and Central Europe |
| Iceland | ISL | Western and Central Europe |
| India | IND | South Asia |
| Indonesia | IDN | East Asia |
| Iran (Islamic Republic of) | IRN | West Asia |
| Iraq | IRQ | West Asia |
| Ireland | IRL | Western and Central Europe |
| Israel | ISR | West Asia |
| Italy | ITA | Western and Central Europe |
| Jamaica | JAM | Latin America |
| Japan | JPN | East Asia |
| Jordan | JOR | West Asia |
| Kazakhstan | KAZ | Eastern Europe and Central Asia |
| Kenya | KEN | Africa |
| Kiribati | KIR | Oceania |
| Kuwait | KWT | West Asia |
| Kyrgyzstan | KGZ | Eastern Europe and Central Asia |
| Lao People’s Democratic Republic | LAO | East Asia |
| Latvia | LVA | Eastern Europe and Central Asia |
| Lebanon | LBN | West Asia |
| Lesotho | LSO | Africa |
| Liberia | LBR | Africa |
| Libya | LBY | Africa |
| Liechtenstein | LIE | Western and Central Europe |
| Lithuania | LTU | Eastern Europe and Central Asia |
| Luxembourg | LUX | Western and Central Europe |
| Madagascar | MDG | Africa |
| Malawi | MWI | Africa |
| Malaysia | MYS | East Asia |
| Maldives | MDV | South Asia |
| Mali | MLI | Africa |
| Malta | MLT | Western and Central Europe |
| Marshall Islands | MHL | Oceania |
| Mauritania | MRT | Africa |
| Mauritius | MUS | Africa |
| Mexico | MEX | Latin America |
| Micronesia (Federated States of) | FSM | Oceania |
| Monaco | MCO | Western and Central Europe |
| Mongolia | MNG | East Asia |
| Montenegro | MNE | Western and Central Europe |
| Morocco | MAR | Africa |
| Mozambique | MOZ | Africa |
| Myanmar | MMR | East Asia |
| Namibia | NAM | Africa |
| Nauru | NRU | Oceania |
| Nepal | NPL | South Asia |
| Netherlands | NLD | Western and Central Europe |
| New Zealand | NZL | Oceania |
| Nicaragua | NIC | Latin America |
| Niger | NER | Africa |
| Nigeria | NGA | Africa |
| North Macedonia | MKD | Western and Central Europe |
| Norway | NOR | Western and Central Europe |
| Oman | OMN | West Asia |
| Pakistan | PAK | South Asia |
| Palau | PLW | Oceania |
| Panama | PAN | Latin America |
| Papua New Guinea | PNG | Oceania |
| Paraguay | PRY | Latin America |
| Peru | PER | Latin America |
| Philippines | PHL | East Asia |
| Poland | POL | Western and Central Europe |
| Portugal | PRT | Western and Central Europe |
| Qatar | QAT | West Asia |
| Republic of Korea | KOR | East Asia |
| Republic of Moldova | MDA | Eastern Europe and Central Asia |
| Romania | ROU | Western and Central Europe |
| Russian Federation | RUS | Eastern Europe and Central Asia |
| Rwanda | RWA | Africa |
| Saint Kitts and Nevis | KNA | Latin America |
| Saint Lucia | LCA | Latin America |
| Saint Vincent and the Grenadines | VCT | Latin America |
| Samoa | WSM | Oceania |
| San Marino | SMR | Western and Central Europe |
| Sao Tome and Principe | STP | Africa |
| Saudi Arabia | SAU | West Asia |
| Senegal | SEN | Africa |
| Serbia | SRB | Western and Central Europe |
| Seychelles | SYC | Africa |
| Sierra Leone | SLE | Africa |
| Singapore | SGP | East Asia |
| Slovakia | SVK | Western and Central Europe |
| Slovenia | SVN | Western and Central Europe |
| Solomon Islands | SLB | Oceania |
| Somalia | SOM | Africa |
| South Africa | ZAF | Africa |
| South Sudan | SSD | Africa |
| Spain | ESP | Western and Central Europe |
| Sri Lanka | LKA | South Asia |
| Sudan | SDN | Africa |
| Suriname | SUR | Latin America |
| Sweden | SWE | Western and Central Europe |
| Switzerland | CHE | Western and Central Europe |
| Syrian Arab Republic | SYR | West Asia |
| Tajikistan | TJK | Eastern Europe and Central Asia |
| Thailand | THA | East Asia |
| North Macedonia | MKD | Western and Central Europe |
| Timor-Leste | TLS | East Asia |
| Togo | TGO | Africa |
| Tonga | TON | Oceania |
| Trinidad and Tobago | TTO | Latin America |
| Tunisia | TUN | Africa |
| Turkey | TUR | West Asia |
| Turkmenistan | TKM | Eastern Europe and Central Asia |
| Tuvalu | TUV | Oceania |
| Uganda | UGA | Africa |
| Ukraine | UKR | Eastern Europe and Central Asia |
| United Arab Emirates | ARE | West Asia |
| United Kingdom of Great Britain and Northern Ireland | GBR | Western and Central Europe |
| United Republic of Tanzania | TZA | Africa |
| United States of America | USA | North America |
| Uruguay | URY | Latin America |
| Uzbekistan | UZB | Eastern Europe and Central Asia |
| Vanuatu | VUT | Oceania |
| Venezuela, Bolivarian Republic of | VEN | Latin America |
| Viet Nam | VNM | East Asia |
| Yemen | YEM | West Asia |
| Zambia | ZMB | Africa |
| Zimbabwe | ZWE | Africa |

### Supplementary Table 2: Number of forms sent out, and responded to in the survey.

| **Country** | **Number of survey forms or requests for information sent out** | **Number of replies returned** | **Data received in required format** | **Number of respondents used in final dataset*** |
| --- | --- | --- | --- | --- |
| Argentina | 1 | 1 | No | Yes |
| Australia | 3 | 0 | No | No |
| Austria | 1 | 1 | No | Yes |
| Bangladesh | 5 | 0 | No | No |
| Belarus | 1 | 1 | Yes | Yes |
| Belgium | 1 | 1 | No | Yes |
| Bolivia | 1 | 1 | Yes | Yes |
| Brazil | 5 | 2 | Yes | Yes |
| Bulgaria | 1 | 1 | No | Yes |
| Burkina Faso | 2 | 2 | No | Yes |
| Canada | 1 | 1 | Yes | Yes |
| Chile | 1 | 1 | Yes | Yes |
| China | 3 | 2 | Yes | Yes |
| Colombia | 1 | 1 | Yes | No |
| Côte D'Ivoire | 1 | 1 | No | No |
| Croatia | 1 | 1 | No | Yes |
| Cyprus | 1 | 1 | No | Yes |
| Czech Republic | 1 | 1 | No | Yes |
| Denmark | 1 | 1 | No | Yes |
| Egypt | 2 | 1 | No | No |
| El Salvador | 1 | 0 | No | No |
| Estonia | 1 | 1 | No | Yes |
| Ethiopia | 2 | 2 | No | No |
| Finland | 1 | 1 | No | Yes |
| France | 2 | 1 | No | Yes |
| Germany | 2 | 2 | No | Yes |
| Greece | 1 | 1 | No | Yes |
| Hungary | 1 | 1 | No | Yes |
| India | 2 | 1 | Yes | Yes |
| Indonesia | 4 | 0 | No | No |
| Iran | 1 | 0 | No | No |
| Ireland | 1 | 1 | No | Yes |
| Israel | 1 | 1 | No | Yes |
| Italy | 1 | 1 | No | Yes |
| Japan | 1 | 1 | Yes | Yes |
| Kenya | 2 | 2 | No | No |
| Latvia | 1 | 1 | No | Yes |
| Lithuania | 1 | 1 | No | Yes |
| Malaysia | 3 | 0 | No | No |
| Mali | 2 | 2 | No | Yes |
| Mexico | 5 | 0 | No | No |
| Morocco | 1 | 0 | No | No |
| Mozambique | 2 | 2 | No | No |
| Myanmar | 2 | 1 | Yes | Yes |
| Nepal | 1 | 0 | No | No |
| Netherlands | 1 | 1 | No | Yes |
| New Zealand | 1 | 1 | No | Yes |
| Nigeria | 3 | 2 | No | Yes |
| Norway | 1 | 1 | No | Yes |
| Pakistan | 3 | 2 | Yes | Yes |
| Paraguay | 1 | 1 | Yes | Yes |
| Peru | 1 | 1 | Yes | Yes |
| Philippines | 3 | 1 | Yes | Yes |
| Poland | 2 | 1 | No | Yes |
| Portugal | 1 | 1 | No | Yes |
| Romania | 1 | 1 | No | Yes |
| Russian Federation | 4 | 0 | No | No |
| Senegal | 2 | 2 | No | Yes |
| Slovakia | 1 | 1 | No | Yes |
| Slovenia | 1 | 1 | No | Yes |
| South Africa | 2 | 1 | Yes | Yes |
| Spain | 1 | 1 | No | Yes |
| Sweden | 1 | 1 | No | Yes |
| Taiwan | 1 | 0 | No | No |
| United Republic of Tanzania | 2 | 2 | No | Yes |
| Thailand | 1 | 1 | Yes | Yes |
| Turkey | 2 | 1 | Yes | Yes |
| Uganda | 2 | 2 | No | No |
| Ukraine | 3 | 1 | No | No |
| United Kingdom of Great Britain and Northern Ireland | 2 | 2 | No | Yes |
| Uruguay | 3 | 2 | Yes | Yes |
| United States of America | 1 | 1 | Yes | Yes |
| Uzbekistan | 1 | 0 | No | No |
| Viet Nam | 4 | 0 | No | No |
| Zambia | 2 | 2 | No | No |
| Zimbabwe | 1 | 1 | No | No |

### Supplementary Table 3: Survey questionnaire template


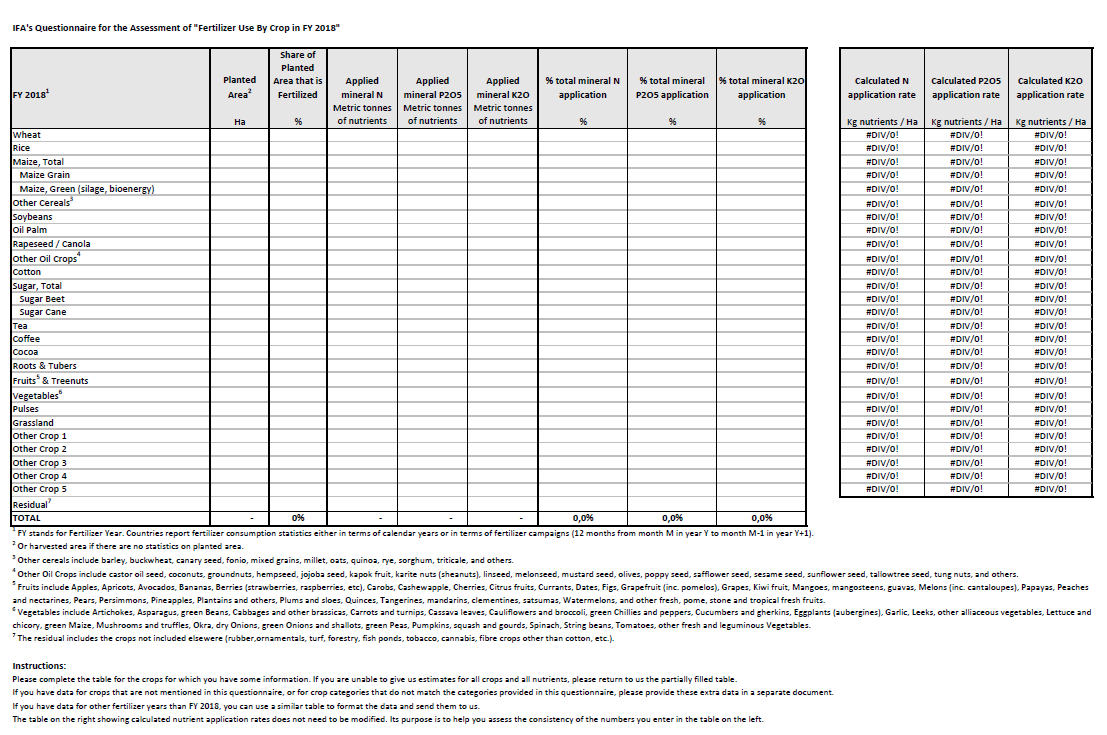


### Supplementary Table 4: Comparison in estimates of the total consumption of inorganic fertilizer (as nitrogen + P_2_O_5_ + K_2_O) by country based on the latest fertilizer use by crop survey (FUBC 9) and from the International Fertilizer Association Statistics (IFASTAT) source.

| **Country** | **FUBC 9 estimate (1000 metric tonnes per country per year)** | **IFASTAT estimate (1000 metric tonnes per country per year)** | **Percentage difference (relative to IFASTAT)** |
| --- | --- | --- | --- |
| Argentina | 1882 | 1882 | 0 |
| Australia | 2493 | 2433 | 2 |
| Austria | 176 | 170 | 4 |
| Belgium | 152 | 268 | -43 |
| Bangladesh | 2446 | 2446 | 0 |
| Bulgaria | 456 | 583 | -22 |
| Belarus | 892 | 892 | 0 |
| Brazil | 15798 | 15507 | 2 |
| Canada | 4172 | 4793 | -13 |
| Chile | 499 | 447 | 12 |
| China | 61937 | 45748 | 35 |
| Cyprus | 8 | 14 | -46 |
| Czech Republic | 441 | 421 | 5 |
| Germany | 2156 | 1953 | 10 |
| Denmark | 322 | 306 | 5 |
| Egypt | 1593 | 1692 | -6 |
| Spain | 1891 | 1882 | 1 |
| Estonia | 87 | 94 | -8 |
| Finland | 200 | 213 | -6 |
| France | 3116 | 3059 | 2 |
| United Kingdom of Great Britain and Northern Ireland | 1482 | 1491 | -1 |
| Greece | 274 | 276 | -1 |
| Croatia | 157 | 194 | -19 |
| Hungary | 554 | 568 | -2 |
| Indonesia | 7125 | 7202 | -1 |
| India | 27228 | 27228 | 0 |
| Ireland | 656 | 660 | -1 |
| Iran (Islamic Republic of) | 1086 | 1088 | 0 |
| Israel | 87 | 89 | -2 |
| Italy | 879 | 876 | 0 |
| Japan | 977 | 934 | 5 |
| Lithuania | 278 | 289 | -4 |
| Latvia | 180 | 168 | 7 |
| Morocco | 434 | 388 | 12 |
| Mexico | 2296 | 2262 | 2 |
| Mali | 256 | 316 | -19 |
| Myanmar | 465 | 465 | 0 |
| Malaysia | 2163 | 2176 | -1 |
| Nigeria | 519 | 734 | -29 |
| Netherlands | 264 | 231 | 14 |
| Norway | 161 | 163 | -2 |
| New Zealand | 782 | 952 | -18 |
| Pakistan | 4557 | 4615 | -1 |
| Peru | 472 | 472 | 0 |
| Philippines | 961 | 1034 | -7 |
| Poland | 2077 | 1906 | 9 |
| Portugal | 127 | 127 | 0 |
| Paraguay | 482 | 296 | 63 |
| Romania | 964 | 1042 | -8 |
| Russian Federation | 3219 | 3547 | -9 |
| Senegal | 88 | 51 | 72 |
| Slovakia | 173 | 204 | -15 |
| Slovenia | 43 | 39 | 12 |
| Sweden | 233 | 245 | -5 |
| Thailand | 2650 | 2264 | 17 |
| Turkey | 2918 | 2184 | 34 |
| United Republic of Tanzania | 112 | 148 | -24 |
| Ukraine | 2151 | 2096 | 3 |
| Uruguay | 300 | 384 | -22 |
| United States of America | 19684 | 20359 | -3 |
| Viet Nam | 2928 | 2940 | 0 |
| South Africa | 763 | 762 | 0 |
